# Bone Morphogenetic Protein-2 and Cabozantinib for Temporomandibular Joint Cartilage Repair: Murine and Porcine Injury Models

**DOI:** 10.3390/ijms27198937

**Published:** 2026-10-08

**Authors:** Max L. Silverstein, Sophia Hou, Chunxi Ge, Tanya C. Garcia, Charles K. F. Chan, Karl C. Bruckman, Eri Takematsu

**Affiliations:** 1 Division of Plastic and Reconstructive Surgery, Department of Surgery, Stanford University School of Medicine, 770 Welch Rd. Ste 400, Stanford, CA 94305, USA; 2 Department of Periodontics and Oral Medicine, School of Dentistry, University of Michigan, 1011 N. University Ave., Ann Arbor, MI 48109, USA; 3 Veterinary Institute for Regenerative Cures, School of Veterinary Medicine, University of California, Davis, 1 Shields Ave., Davis, CA 95616, USA

**Keywords:** temporomandibular joint, osteoarthritis, cartilage, fibrocartilage, regeneration, bone morphogenetic protein-2, cabozantinib, large animal model, tissue engineering

## Abstract

Temporomandibular joint osteoarthritis (TMJ OA) is characterized by progressive degeneration of the fibrocartilaginous articular surface. We previously demonstrated that combined bone morphogenetic protein-2 (BMP-2) and vascular endothelial growth factor (VEGF) inhibition promotes endogenous hyaline cartilage regeneration in appendicular joints. Whether this regenerative strategy can be extended to the specialized fibrocartilage of the TMJ remains unknown. In this study, we evaluated the regenerative potential of BMP-2 combined with cabozantinib, an FDA-approved multikinase inhibitor with activity against VEGF receptors, using complementary murine and porcine TMJ cartilage injury models. In mice, needle-induced abrasion of the mandibular condyle resulted in loss of the fibrocartilaginous articular surface and extracellular matrix; treatment with BMP-2 and cabozantinib was associated with improved cartilage architecture and increased fibrocartilage thickness. To examine translational feasibility, we developed a surgically accessible porcine TMJ cartilage defect model and demonstrated the technical feasibility of local BMP-2 and cabozantinib delivery with formation of repair tissue within the defect. Collectively, these findings provide preliminary evidence supporting further investigation of combined BMP-2 and cabozantinib for TMJ cartilage repair.

## 1. Introduction

Temporomandibular disorders (TMDs) represent a common and debilitating condition affecting approximately 5–12% of adults and are frequently associated with pain, impaired mastication, limited jaw mobility, and diminished quality of life [1]. Among TMDs, temporomandibular joint osteoarthritis (TMJ OA) is characterized by progressive degeneration of the articular cartilage, remodeling of subchondral bone, and chronic inflammation of surrounding tissues. Similar to osteoarthritis in appendicular joints, TMJ OA results from a multifactorial interplay among mechanical stress, inflammation, aging, genetic susceptibility, and altered cellular homeostasis [2,3,4]. Despite the prevalence of TMJ degeneration, currently available treatments remain largely palliative and focus primarily on symptom management through analgesics, physical therapy, occlusal modification, or surgical intervention in advanced cases [3]. Therapies capable of restoring damaged TMJ cartilage remain limited.

The development of regenerative approaches for cartilage repair has emerged as an important objective in musculoskeletal medicine. Growth factor-based therapies are particularly attractive due to their ability to stimulate endogenous repair pathways while avoiding many of the translational and regulatory challenges associated with cell-based or gene-based therapeutics [5,6]. Previously, our group demonstrated that activation of native skeletal stem cells through combined administration of bone morphogenetic protein-2 (BMP-2) and vascular endothelial growth factor (VEGF) inhibition promoted regeneration of hyaline cartilage in articular knee defects [7]. In a subsequent preclinical porcine study, we demonstrated that BMP-2 combined with cabozantinib, an FDA-approved multikinase inhibitor with activity against VEGF receptors, similarly enhanced hyaline cartilage regeneration in the knee [8]. Cabozantinib blocks the phosphorylation of VEGFR2, thereby inhibiting angiogenesis of endothelial cells and creating an avascular environment for cartilage formation [9]. Cabozantinib also promoted chondrogenic differentiation of skeletal stem cells through modulation of EGFR–ERK signaling, suggesting that its potential effects on cartilage repair may involve multiple mechanisms [10]. These findings suggest that coordinated modulation of osteochondral repair pathways using a growth factor and a small-molecule inhibitor can direct endogenous progenitor cells toward functional cartilage regeneration.

However, whether this regenerative strategy can be successfully adapted to the temporomandibular joint remains unknown. In contrast to the knee joint, the mandibular condyle is covered predominantly by fibrocartilage rather than hyaline cartilage, possessing unique structural and biomechanical properties suited to repetitive loading and mandibular movement [3,11]. Consequently, regenerative approaches optimized for appendicular joints may produce distinct repair phenotypes in the TMJ microenvironment. Moreover, given the osteogenic activity of BMP-2 and the broad kinase-inhibitory profile of cabozantinib, the combination of factors could carry risks to the TMJ osteochondral environment, such as heterotopic ossification, subchondral bone remodeling, and impaired vascular healing. Understanding whether BMP-2 and cabozantinib can induce TMJ-appropriate cartilage regeneration with limited off-target effects, therefore, represents an important unanswered question.

Preclinical investigation of TMJ regeneration is further limited by the availability of suitable animal models [12]. Murine models have primarily been used to investigate the molecular mechanisms underlying TMJ development and disease through genetic manipulation [13,14], whereas direct cartilage injury models remain technically challenging because of the small size of the joint. In contrast, porcine models closely recapitulate the anatomy, biomechanics, and surgical accessibility of the human TMJ, allowing creation of clinically relevant cartilage defects and evaluation of regenerative intervention [15,16]. However, regenerative strategies have not been systematically evaluated across both murine and porcine TMJ injury models.

In the present study, we utilized complementary murine and porcine TMJ cartilage injury models to evaluate the regenerative potential of combined BMP-2 and cabozantinib treatment. In the murine model, we assessed cartilage repair following needle-induced abrasion of the mandibular condyle using histological and matrix-specific analyses. We then performed a pilot study using a surgically accessible porcine TMJ defect model to examine the feasibility of local therapeutic delivery in a clinically relevant large-animal platform. Together, these complementary models provide a translational framework for investigating regenerative therapies for TMJ cartilage repair and support future validation of this approach in larger preclinical studies.

## 2. Results

### 2.1. BMP-2 and Cabozantinib Treatment Is Associated with Histological Changes Consistent with Cartilage Repair in a Murine TMJ Injury Model

**Figure 1:**
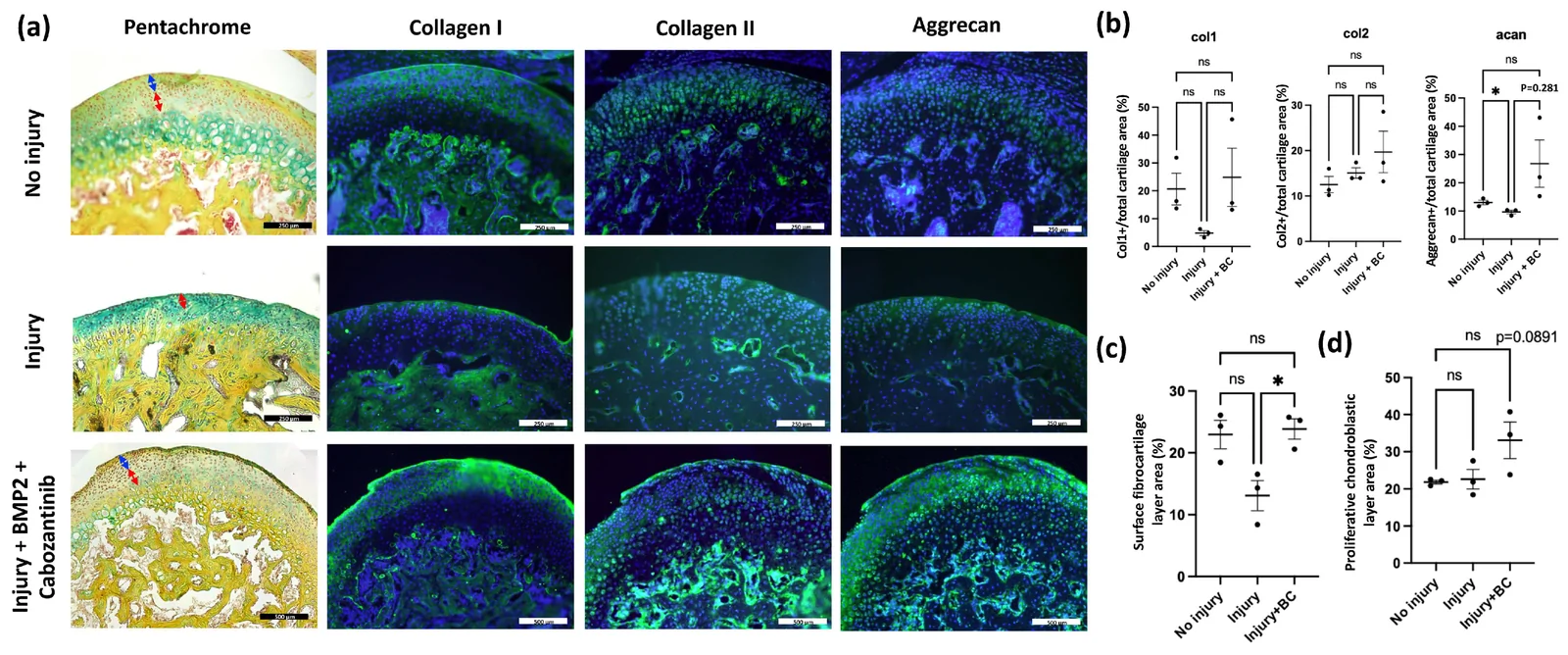
Histological and immunofluorescence evaluation of TMJ cartilage repair following BMP-2 and cabozantinib treatment in the murine TMJ injury model: (**a**) Representative pentachrome staining and immunofluorescence staining for collagen I, collagen II, and aggrecan in the mandibular condyle from the no-injury, injury, and injury + treatment groups. Pentachrome staining demonstrates loss of the superficial fibrocartilaginous layer (blue arrow) and thinning of the proliferative chondroblastic layer (red arrow) following injury. Treatment with BMP-2 and cabozantinib was associated with partial recovery of the fibrocartilaginous layer and increased extracellular matrix deposition. Scale bar is 250 µm for no injury and injury group and 500 µm for injury + BMP2 + Cabozantinib group. (**b**) Quantification of collagen I-positive fibrocartilage area, collagen II-positive cartilage area, and aggrecan-positive cartilage area normalized to the total cartilage area. (**c**) Fibrocartilage layer area relative to total cartilage area was quantified from pentachrome staining, demonstrating a reduction following injury and an increase following BMP-2 and cabozantinib treatment. (**d**) Proliferative chondroblastic layer area relative to total cartilage area was quantified from pentachrome staining, demonstrating a numerical increase following BMP-2 and cabozantinib treatment. Statistical significance was determined by one-way ANOVA with Tukey’s multiple-comparison test. Data are presented as mean ± SEM. *, *p* < 0.05; ns, not significant. *n* = 3 biological replicates.

To evaluate the regenerative potential of BMP-2 and cabozantinib in the temporomandibular joint, we established a murine TMJ cartilage abrasion model in which focal condylar injury was followed by intra-articular administration of PBS or BMP-2 + cabozantinib. TMJs were harvested three weeks after treatment for histological and immunofluorescence analyses. Histological examination of uninjured condyles demonstrated an organized fibrocartilaginous articular surface with preserved thickness and cellular organization (Figure 1a). Pentachrome staining revealed a continuous fibrocartilage layer (blue arrow) overlying a well-defined proliferative chondroblastic layer (red arrow) (Figure 1a). Histological evaluation further demonstrated no evidence of heterotopic ossification or subchondral bone remodeling in this pilot study. Immunofluorescence staining demonstrated prominent collagen I expression within the fibrocartilage layer, whereas collagen II and aggrecan were predominantly localized to the proliferative chondroblastic layer (Figure 1a). In contrast, injured condyles exhibited thinning of the fibrocartilage layer and a reduced proliferative chondroblastic layer (red arrow) (Figure 1a). Quantitative analysis demonstrated a significant reduction in aggrecan-positive cartilage area compared with uninjured controls, while collagen I- and collagen II-positive areas were not significantly altered (Figure 1b). Treatment with BMP-2 and cabozantinib improved the overall cartilage architecture following injury. Pentachrome staining demonstrated increased cartilage thickness and expansion of matrix-rich tissue at the condylar surface (Figure 1a). Both the fibrocartilage layer (blue arrow) and proliferative chondroblastic layer (red arrow) appeared thicker than in untreated injured condyles, consistent with improved cartilage architecture (Figure 1a). Immunofluorescence staining showed increased aggrecan and collagen II staining within the proliferative chondroblastic layer and collagen I staining within the fibrocartilage layer; however, these differences were not statistically significant by quantitative analysis (Figure 1b). We quantified the fibrocartilage and proliferative chondroblastic layer area relative to the total cartilage area using pentachrome-stained sections. The relative fibrocartilage layer area was reduced following injury and increased following BMP-2 and cabozantinib treatment (Figure 1c). Similarly, the relative area of the proliferative chondroblastic layer was increased following BMP-2 and cabozantinib treatment compared with the control group, although the difference did not reach statistical significance (Figure 1d). Together, these quantitative analyses are consistent with the histological observation of partial recovery of fibrocartilage and proliferative chondroblastic layers following treatment. Collectively, these findings indicate that BMP-2 and cabozantinib improved the structural organization of the injured TMJ condyle and were associated with partial recovery of the fibrocartilage and proliferative chondroblastic layers, although quantitative changes in extracellular matrix markers did not reach statistical significance.

### 2.2. Development of a Surgically Accessible Porcine TMJ Defect Model

**Figure 2:**
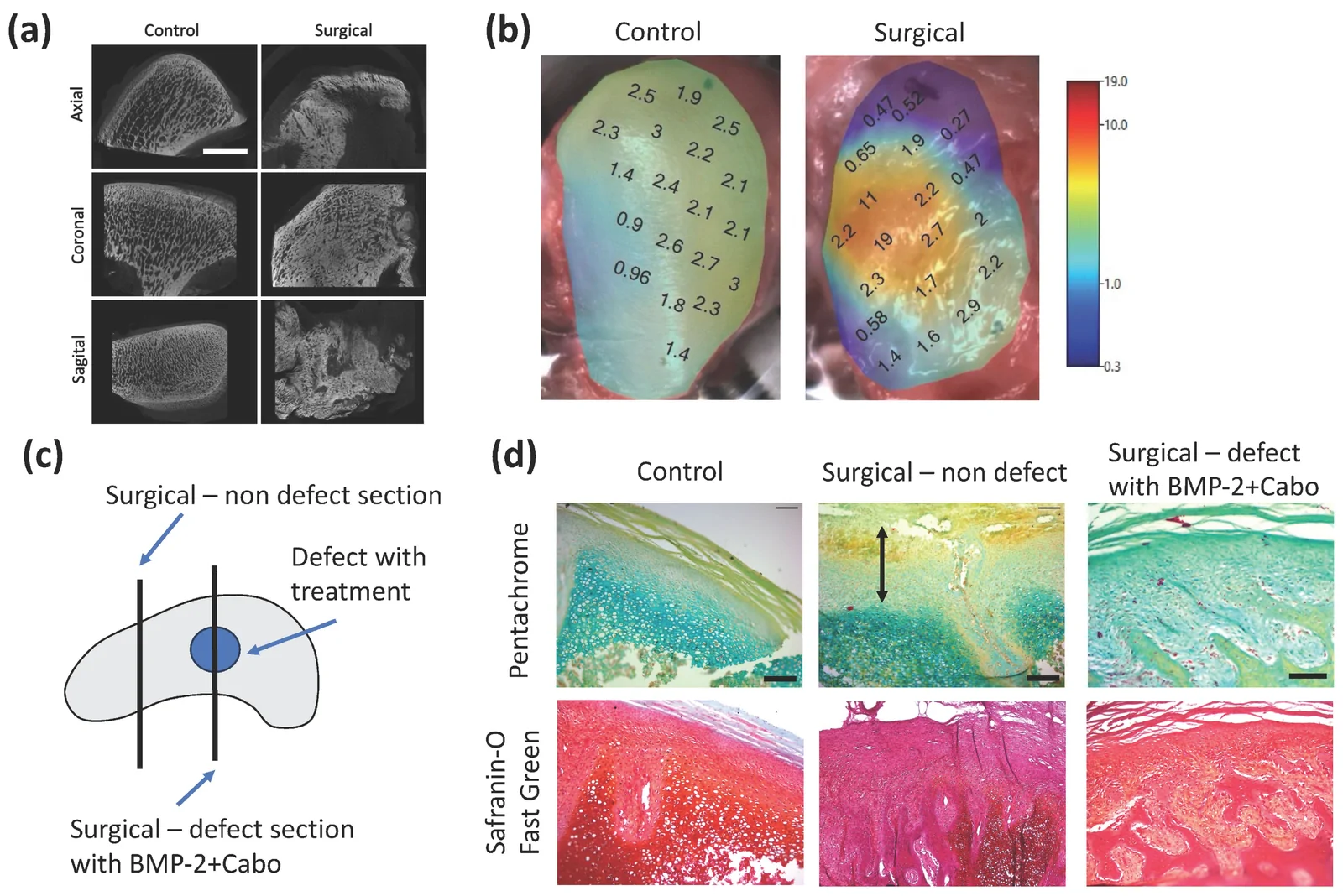
Characterization of the porcine TMJ defect model and histological evaluation following BMP-2 and cabozantinib treatment: (**a**) Representative micro-computed tomography (micro-CT) images of the control and surgically treated TMJ condyles. Scale bar = 1 cm. (**b**) Heat maps illustrating the distribution of Young’s instantaneous elastic modulus across the condylar head in the control and surgically treated TMJs (MPa). (**c**) Schematic illustrating the histological sectioning planes. The surgical non-defect plane was obtained from the surgically treated condyle outside the defect region, whereas the defect + treatment plane corresponded to the defect site implanted with a scaffold containing BMP-2 and cabozantinib. (**d**) Representative pentachrome- and safranin O/fast green-stained sections of the control condyle, the surgical non-defect region, and the defect region treated with a BMP-2- and cabozantinib-loaded scaffold. Scale bar = 100 µm. Black arrows indicate the thickness of the fibrocartilaginous layer.

To facilitate future translational studies, we performed a large-animal porcine model enabling direct surgical access to the mandibular condyle and placement of regenerative scaffolds. Briefly, we created defects on the condylar cartilage surface using a 4 mm biopsy punch, into which the circular 4 mm scaffolds containing BMP-2 and cabozantinib were placed. Micro-computed tomography demonstrated the disruption of subchondral architecture in the surgically manipulated joint relative to the uninjured control, consistent with the surgically created defect/injury (Figure 2a).

Subsequent biomechanical analysis assessing the Young’s instantaneous elastic modulus of the condylar head (Figure 2b) indicated a change in the stiffness of the articular surface following surgery. The modulus of the control condylar surface was measured at 2.06 ± 0.14 MPa (mean ± SD across 19 indentation positions on the single condyle) (Figure 2b control), with slightly greater stiffness on the buccal aspect compared to the lingual side. In contrast, the modulus on the surgical side was higher and more irregular overall, at 2.95 ± 1.04 MPa, with especially high stiffness at the center of the articular surface (Figure 2b surgical). The modulus at the periphery of the surgical condyle was lower compared to the more uniform surface of the control.

Following CT examination, TMJ samples were sectioned for histological analysis. On the surgical side, both the non-defect and defect planes were examined for evaluation (Figure 2c). Histochemical staining of the control (non-injured) condylar head revealed a smooth and organized fibrocartilage layer (stained white-brown) on top of a layer of healthy-appearing chondrocytes (stained dark blue) (Figure 2d, control). In comparison, the articular surface of the surgical side formed a thick layer of fibrocartilage (black arrow) on densely packed chondrocytes (Figure 2c, surgical non-defect). We also sectioned and stained the site of defect creation and treatment application on the condylar joint surface, which showed an aggrecan-enriched fibrocartilage layer within the defect following BMP-2 and cabozantinib-containing scaffold placement, as indicated by alcian blue staining (Figure 2c, surgical defect with BMP-2 and cabozantinib). Preliminary observations demonstrated structural and biomechanical alterations of the condylar surface following surgical injury, together with the technical feasibility of local BMP-2 and cabozantinib delivery via scaffold implantation in a clinically relevant large-animal model. In the absence of an untreated defect control, the observed repair tissue cannot be attributed specifically to the treatment. Collectively, these findings support the feasibility of this porcine TMJ defect model for future controlled studies of cartilage repair and regenerative therapies.

## 3. Discussion

In this study, we demonstrate that combined BMP-2 and cabozantinib treatment is associated with histological trends consistent with cartilage repair in a mouse temporomandibular joint injury model and provide proof of concept for a surgically accessible porcine cartilage defect platform for future translational studies. Our findings extend previous observations from appendicular joints and suggest that regenerative signaling through BMP-2 and cabozantinib treatment may be adapted to the distinct fibrocartilaginous environment of the mandibular condyle.

A major finding of this study is that TMJ cartilage repair appears fundamentally distinct from previously reported knee regeneration. In prior work, combined BMP-2 and VEGF inhibition promoted regeneration of hyaline cartilage in knee defects [7,8]. In contrast, the repair tissue observed in the mouse TMJ exhibited features of both fibrocartilage and the proliferative chondroblastic layer (Figure 1b), characterized by preservation of collagen I expression together with numerically increased aggrecan and collagen II deposition. These findings likely reflect differences in native tissue composition between joints. Importantly, the cell signaling basis for the response we observed in the TMJ, including the roles of canonical BMP signaling, VEGF/VEGFR pathway modulation, and recruitment of skeletal stem/progenitor cells, was not examined in the present model. Direct molecular and cellular investigation of these pathways in the TMJ represents an important direction for future work.

Unlike articular knee cartilage, the TMJ condyle is covered by fibrocartilage adapted to repetitive multidirectional loading [17]. Fibrocartilage provides greater resistance to shear and mechanical stress while maintaining chondrogenic properties necessary for joint function [17]. Consequently, successful TMJ cartilage repair may not require recreation of pure hyaline cartilage but instead restoration of a fibrocartilaginous matrix appropriate for mandibular biomechanics [18]. Our findings therefore suggest that BMP-2 and cabozantinib may be associated with context-dependent histological changes consistent with cartilage repair.

The murine model served as an important platform for evaluating treatment-associated histological changes and tissue phenotype. Histological and immunofluorescence findings demonstrated qualitative changes consistent with cartilage matrix repair following injury and BMP-2 + cabozantinib treatment, in addition to quantitative trends toward increased aggrecan and collagen II deposition (Figure 1a,b). An increase in collagen I expression in the fibrocartilage layer of the treatment group toward uninjured levels, though not statistically significant, was similarly encouraging given the specialized structure of the mandibular condyle (Figure 1a,b). These observations support the hypothesis that regenerative therapies may require joint-specific evaluation rather than assuming conserved outcomes across anatomical sites.

To facilitate future translational research, we also developed a surgically accessible porcine TMJ model that allows direct injury and simultaneous scaffold implantation at the condylar surface. Existing porcine TMJ OA models commonly rely on disc perforation or discectomy to induce secondary joint degeneration over time [19,20]. While these models reproduce progressive osteoarthritic changes, they do not generate a localized osteochondral defect that readily accommodates implantation or retention of regenerative biomaterials. In contrast, our approach enables precise creation of condylar defects while providing immediate access for therapeutic delivery. This technique aligns with emerging clinical evidence for local biomaterial-based approaches to osteoarthritis; a recent study of intra-articular carboxymethyl–chitosan injection in patients with knee osteoarthritis reported significant short-term improvements in pain and function, though the relevance of these findings to TMJ fibrocartilage requires investigation [21].

Importantly, the porcine TMJ shares key anatomical and biomechanical characteristics with the human TMJ, making it a valuable translational platform for evaluating regenerative interventions [12,15]. The porcine model additionally enabled characterization of biomechanical changes associated with TMJ degeneration. Altered Young’s instantaneous elastic modulus across injured condyles suggests substantial changes in tissue mechanical integrity following cartilage disruption (Figure 2b). Such biomechanical analyses may provide useful benchmarks for evaluating future regenerative success, as restoration of tissue composition alone may not necessarily correspond to restoration of function. Future studies should, therefore, also report functional outcome measures such as jaw kinematics, bite force, or mastication behavior to determine whether condylar cartilage repair translates into restored TMJ function.

Several limitations warrant consideration. (1) While animals were randomized to treatment groups, histological and immunofluorescence image acquisition and analysis were performed without blinding to group assignment, which may have introduced bias in the qualitative interpretation of the murine data. (2) Although quantitative analyses of fibrocartilage and proliferative chondroblastic layer areas and matrix-associated markers (collagen I, collagen II, and aggrecan) were performed, the histological assessment remains limited by the small sample size and the lack of more detailed quantitative evaluation of cartilage thickness and cellular organization. Future studies with larger cohorts and additional histomorphometric analyses will be necessary to more comprehensively characterize treatment-associated cartilage repair. (3) To minimize biological variability, only male animals were used in this study, but this design limits the generalizability of our findings to female animals. (4) Longer-term studies are crucial to determine whether the observed repair tissue persists, matures, integrates with native cartilage, and maintains mechanical properties over time. (5) The factor concentrations used in this study were based on previous work in the knee rather than TMJ-specific data [10]. Dedicated dose–response and toxicity studies are necessary to establish the pharmacokinetics, safety, and long-term efficacy of local BMP-2 and cabozantinib administration in the TMJ before this approach can be considered for clinical translation. The individual contributions of each factor, and whether their combination acts synergistically in the TMJ, cannot be determined from this study design. (6) Systemic exposure to BMP-2 and cabozantinib following local TMJ administration was not evaluated. Although no overt discomfort or abnormal behavior attributable to treatment was observed in mice, systemic drug concentrations and treatment-related local cellular toxicity were not specifically assessed in the present study. Future studies should evaluate local tissue toxicity, effects on resident joint cells, and systemic exposure following TMJ delivery, particularly when translating this approach to larger animals or clinical applications. (7) The porcine TMJ defect model presents unique technical challenges because the surgical site is located in close proximity to the oral cavity, increasing the risk of postoperative infection. Improved perioperative infection control and surgical management will be essential for reliably establishing this model and enabling future large-cohort studies. (8) An additional limitation of the porcine pilot study is the absence of an untreated defect control. The large-animal experiment was intended to demonstrate the technical feasibility of surgical defect creation, local therapeutic delivery, and histological evaluation rather than to establish therapeutic efficacy. Future studies incorporating untreated and scaffold-only control defects in larger cohorts will be necessary to determine therapeutic efficacy.

## 4. Materials and Methods

### 4.1. Study Design

**Figure 3:**
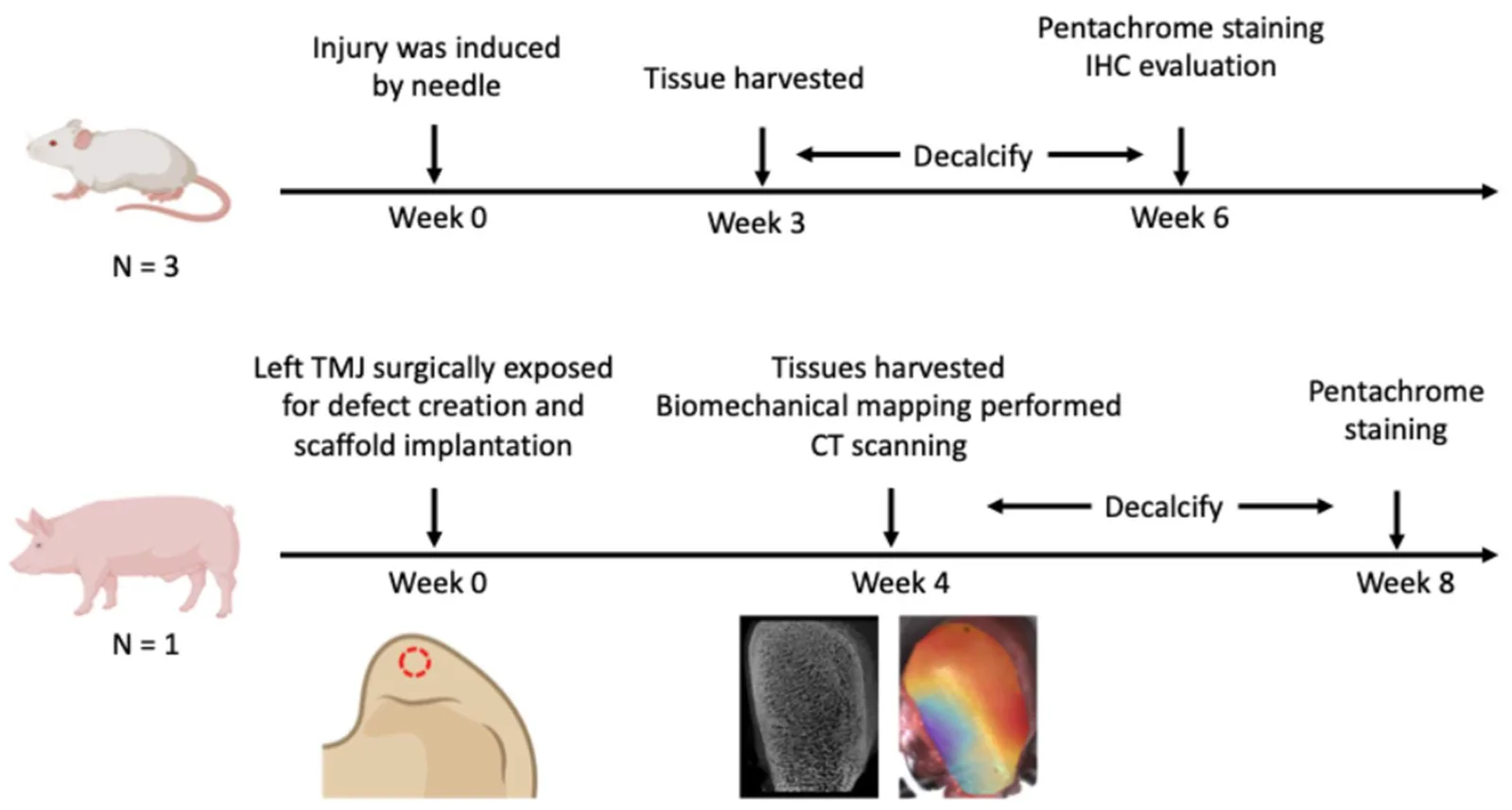
Study design. (**Top**): Mouse study design. TMJ injury was induced by needle abrasion. Three weeks after injury, tissues were harvested and decalcified for 3 weeks, followed by pentachrome staining and immunofluorescence evaluation (*n* = 3 biological replicates per group). (**Bottom**): Mini-pig study design. The left TMJ was surgically exposed for defect creation and scaffold implantation (location represented by red dash-circle), while the right TMJ served as the control. Four weeks postoperatively, TMJs were harvested for biomechanical evaluation and CT imaging. Following 4 weeks of decalcification, TMJs were sectioned for pentachrome staining and histological evaluation (*n* = 1 biological replicate).

In this study, we utilized 10-week-old male C57BL/6 (B6) mice and an 18-month-old male Yucatan mini-pig that had reached skeletal maturity. For the murine study, a surgically induced TMJ injury model was established, followed by local delivery of treatment into the intra-articular space. Mice were assigned to three groups (*n* = 3 per group; 9 animals total)—uninjured controls, injury + PBS, and injury + BMP-2/cabozantinib—using a randomized allocation scheme. Injury and treatment were applied to both TMJs of each animal within a given group. The individual animal served as the experimental unit for statistical analysis. TMJs were harvested at 3 weeks postoperatively, decalcified in 0.4 M EDTA for three weeks, sectioned, and evaluated using pentachrome staining and immunofluorescence (Figure 3).

For the mini-pig model, a surgical procedure was performed on the left TMJ to create defects in the condylar cartilage, into which factor-containing scaffolds were placed, while the untreated contralateral TMJ served as the control (*n* = 1). An untreated defect control was not included in this pilot feasibility study. Four weeks after surgery, the mini-pig was euthanized, and bilateral TMJs were harvested. Modulus mapping was performed immediately on freshly excised tissues to preserve biomechanical integrity and minimize postmortem changes. Subsequently, micro-computed tomography (micro-CT) scans were obtained. Samples were then decalcified in 0.4 M EDTA for four weeks, embedded in optimal cutting temperature (OCT) compound, sectioned, and processed for histological analysis. Pentachrome staining was performed on three regions of interest: the control condyle, the surgical condyle distant from the defect site, and the surgical condyle at the site of defect creation and scaffold implantation (Figure 3).

Different treatment approaches were used across the two models: BMP-2 and cabozantinib were delivered by direct intra-articular injection in mice, whereas an alginate hydrogel scaffold disc was surgically placed in the mini-pig TMJ. This difference reflects size constraints of the murine TMJ, which precludes mechanical implantation of a solid scaffold disc as performed in the porcine model.

### 4.2. Surgical Procedure (Mouse Model)

Survival surgery was performed on 10-week-old C57BL/6J mice obtained from Jackson Lab (catalog number 000664, Bar Harbor, ME, USA). Anesthesia was induced using 3–4% inhaled isoflurane and maintained using a nose cone delivering 1–2% inhaled isoflurane. Ophthalmic ointment was applied to prevent corneal drying, and animals were maintained on a heat source throughout the procedure to prevent hypothermia. The surgical site was shaved and aseptically prepared. Absence of toe-pinch withdrawal reflex was confirmed before proceeding.

The TMJ was located by palpation and anatomical landmarks. A sterile 25G hypodermic needle was inserted percutaneously into the TMJ articular space until it contacted the temporal fossa bone. The needle was then withdrawn slightly and used to probe the mandibular condyle surface. The tip of the needle was then used to thoroughly abrade the articular surface of the condyle, applying 5 strokes across the cartilage surface under consistent manual pressure. All procedures were performed by a single surgeon (M.S.) to minimize inter-animal variability in injury location and severity; however, injury depth and lesion consistency were not independently confirmed on a per-animal basis.

Recombinant human BMP-2 (rhBMP-2) was obtained from the INFUSE™ Bone Graft (Medtronic, Minneapolis, MN, USA, catalog number 7510050). The lyophilized rhBMP-2 was reconstituted in sterile water. Cabozantinib was initially prepared as a 100 mM stock in DMSO and subsequently diluted into PBS to a nominal concentration of 1 mM (final DMSO concentration, 1%). A fine colloidal suspension was observed during preparation; however, the formulation remained visually homogeneous without obvious macroscopic aggregation. Following cartilage abrasion, 10 µL of vehicle control (PBS containing 1% DMSO; injury group) or 10 µL of PBS containing 1% DMSO, BMP-2 (100 µg/mL), and cabozantinib (1 mM) (injury + treatment group) was injected into the TMJ joint space. Each treated TMJ received 1 µg of BMP-2 and 10 nmol of cabozantinib. Because of the limited aqueous solubility of cabozantinib, the reported concentration represents the nominal formulation concentration, and the dissolved cabozantinib concentration was not determined. This concentration was selected based on our previous in vivo dose–response study, in which cabozantinib concentrations of 1–10 mM promoted cartilage formation in combination with BMP-2, whereas concentrations below 1 mM were associated with BMP-2-induced bone formation. Therefore, 1 mM, the lowest concentration associated with cartilage formation in the previous study, was selected for direct intra-articular delivery in the present study.

Following surgery, animals were closely monitored during recovery from anesthesia and postoperatively for signs of infection, swelling, or distress, with early euthanasia criteria applied as needed. Postoperative analgesia was provided with buprenorphine (0.1 mg/kg, subcutaneous, every 8–12 h for 48–72 h).

### 4.3. Scaffold Generation for Minipig Study

Sodium alginate solution was used to deliver growth factors in the form of a semisolid hydrogel due to its excellent cytocompatibility [22]. A 4% (*w*/*v*) sodium alginate stock solution was prepared by dissolving 40 mg of sodium alginate powder in 1 mL of sterile distilled water overnight. For each scaffold, 6 µL of BMP-2 solution (Medtronic, Minneapolis, MN, USA, catalog number 7510050, 1 mg/mL), 6 µL of 4% sodium alginate solution, and 1.33 µL of cabozantinib stock solution (100 mM; MedChemExpress (Monmouth Junction, NJ, USA), HY-13016) were mixed, resulting in a total volume of approximately 13.3 µL. Each scaffold therefore contained 6 µg of BMP-2 and 133 nmol of cabozantinib in alginate gel, corresponding to a nominal cabozantinib concentration of approximately 10 mM. A fine colloidal suspension was observed during preparation; however, the formulation remained visually homogeneous without obvious macroscopic aggregation. The nominal cabozantinib concentration of 10 mM was selected to be consistent with our previous porcine knee cartilage regeneration study [10]. The mixture was placed between a glass slide and coverslip separated by 1 mm, and 1% CaCl_2_ solution was applied to cross-link the alginate for 1 h. The resulting scaffolds were circular, approximately 4 mm in diameter and 1 mm thick. The release kinetics of BMP-2 and cabozantinib from the alginate scaffold were evaluated in our previous study [10]. Briefly, BMP-2 exhibited sustained release over approximately 10 days, whereas cabozantinib exhibited sustained release over approximately 28 days [10].

### 4.4. Surgical Procedure (Minipig Model)

Gaining surgical access to the pig TMJ can be challenging due to its location, namely behind the zygomatic arch and temporal bone, and depth, below abundant subcutaneous fat and masseter muscle. It is therefore essential for researchers to have a thorough understanding of the relevant anatomy and experience performing the dissection on cadaveric specimens prior to embarking on live pig TMJ surgery.

**Figure 4:**
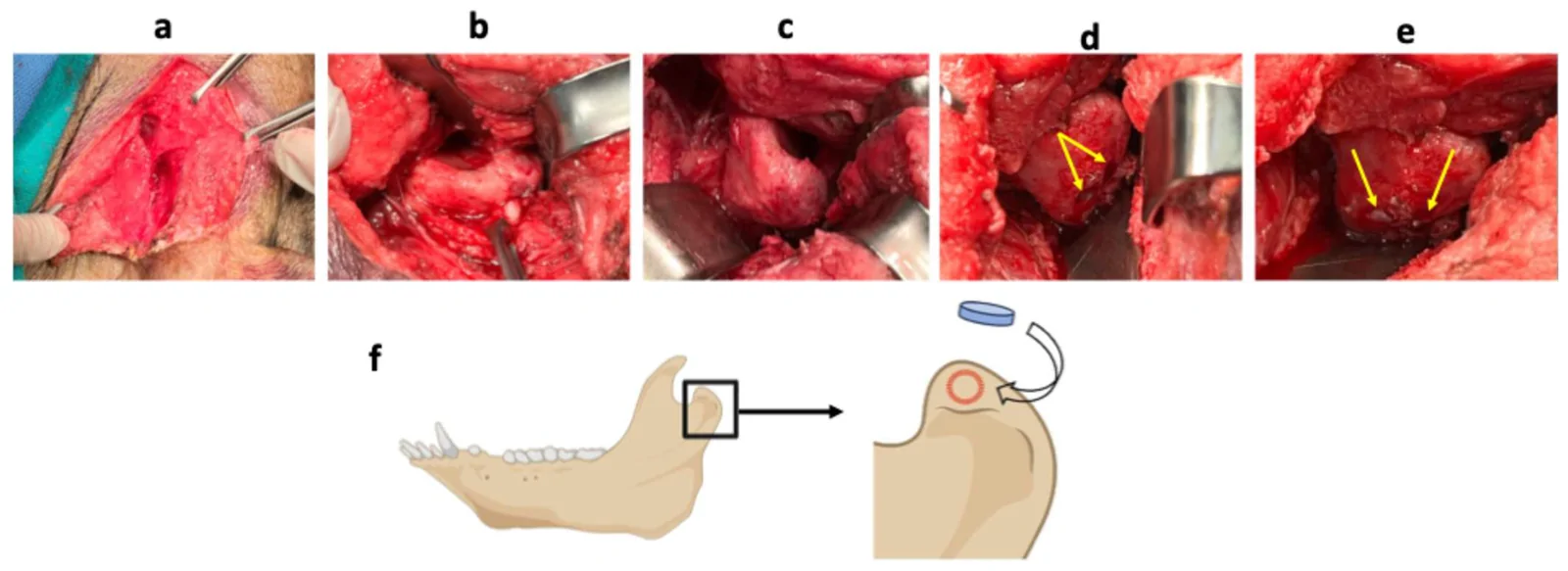
Surgical workflow for porcine TMJ osteochondral defect creation and scaffold implantation: (**a**) Dissecting through subcutaneous fat. (**b**) The masseter muscle was encountered and divided longitudinally with electrocautery. Dissection continued until the TMJ capsule was visualized and opened, revealing the condyle. (**c**) A retractor was then placed against the lingual surface of the ramus to lever the condyle out of the joint and allow for direct inspection of the articular surface. (**d**) Afterward, 4 mm defects were created using a biopsy punch (yellow arrows). (**e**) Scaffolds containing BMP-2 and cabozantinib were carefully placed within the created defects (yellow arrows). (**f**) Schematic illustration of the porcine surgical model. Scaffolds were placed within defects (represented by red circle) created in the cartilage of the condylar head.

In our case, an 18-month-old male Yucatan minipig was obtained from Premier Biosource (Ramona, CA, USA) and housed at the Stanford University Veterinary Service Center (VSC). General anesthesia was induced by VSC staff, and the animal was orotracheally intubated. The TMJ was palpated by opening and closing the mouth with a finger 1–2 cm inferior to the tragus. This location was marked, and an 8 cm line was drawn along the mandibular border and extending retroauricularly, centered on this point. A combination of scalpel and electrocautery was used to dissect directly down toward the TMJ, frequently confirming the trajectory of dissection by opening and closing the mouth while feeling for the joint (Figure 4a). After dissecting through subcutaneous fat, the masseter muscle was encountered and divided longitudinally with electrocautery, revealing the mandibular ramus. A periosteal elevator was used to bluntly elevate the masseter away from the periosteum of the superior mandibular ramus until the TMJ capsule was visualized. A transverse incision was used to open the joint capsule and gain access to the condylar head and glenoid fossa (Figure 4b).

A retractor was then placed against the lingual surface of the ramus to lever the condyle out of the joint and allow for direct inspection of the articular surface (Figure 4c). At this point, two adjacent cartilage defects were created near the center of the condylar articular surface using a 4 mm biopsy punch (Figure 4d). The two defects were initially intended to provide technical replication within the same joint, and both received BMP-2/cabozantinib-loaded scaffolds. At the histological endpoint, however, the two defects had merged into a single continuous defect region and were therefore evaluated as a single treated site. The condyle was then carefully lowered back into the joint and retractors removed, taking care to avoid displacement of the scaffolds. After obtaining meticulous hemostasis, the joint capsule, masseteric fascia, deep dermis, and skin were closed in separate layers. A schematic illustration of the minipig mandible and the location of the scaffold insertion point is shown in Figure 4f. This procedure was performed on only one TMJ per animal, with the contralateral joint serving as an untreated control.

Postoperative analgesia was provided with buprenorphine (0.01–0.03 mg/kg, intramuscular, every 6–12 h) and meloxicam (0.4 mg/kg, subcutaneous or oral, once daily) for several days following surgery. Following surgery, animals were fed a regular diet, and there were no reports of anorexia or apparent pain with mastication. The minipig was euthanized 4 weeks after the operation.

### 4.5. Ethical Approval

We have complied with all relevant ethical regulations. All surgical procedures on mice (Protocol 34757) and minipigs (Protocol 34226) have been approved by Stanford’s Administrative Panel on Laboratory Animal Care. The mouse surgery procedure has also been approved by the University of Michigan Institutional Animal Care & Use Committee (IACUC) under a protocol (PRO00012879).

### 4.6. Elastic Modulus Measurement

The instantaneous elastic modulus was measured to quantify the stiffness of the condyle tissue. Samples were placed in a phosphate buffer solution (PBS) bath at room temperature on a multiaxial mechanical tester equipped with a spherical indenter attached to its load cell (Mach-1v500css, Biomomentum Inc., Laval, QC, Canada). A grid of 19 positions was defined by superimposing locations over the image of the surface to be tested using an integrated video camera. At each position, the tester was programmed to perform an indentation perpendicular to the surface using a 1 mm spherical indenter to a depth of 0.2 mm (approximately equivalent to 20% strain), with an indentation velocity of 0.2 mm/s, held for 5 s, then unloaded.

The indentation was immediately followed by measurement of the cartilage thickness at each position. The thickness was measured by the attachment of a 25-gauge ½” needle that penetrated the cartilage through to bone contact at 0.5 mm/s to a limit of 4 N. Displacement was recorded between contact with the cartilaginous surface (1.0 N) and bone, as detected by a change in stiffness on the load–displacement curve. Displacement between the surface and bone, therefore, represented the thickness (mm) of the cartilage layer. The Mach-1 Analysis Software program (Biomomentum Inc., Laval, QC, Canada) was used to calculate the instantaneous elastic modulus using the curve-fit of load–displacement from the indentation data and the cartilage thickness according to the Hayes model for a spherical indenter [23]. The Hayes model assumes an isotropic, homogeneous, linearly elastic material bonded to a rigid substrate; as TMJ fibrocartilage is anisotropic, this assumption may not be fully met, and modulus values should be interpreted as relative measures rather than absolute material properties. The Mach-1 software generates a map of elastic modulus and thickness by interpolating between positions and extrapolating the surface boundary.

### 4.7. Micro-Computed Tomography (CT)

TMJ condyles were scanned using a Bruker Skyscan 1276 (Bruker Preclinical Imaging, Kontich, Belgium) with a source voltage of 85 kV, a source current of 200 μA, a filter setting of AI 1 mm, and a pixel size of 12 μm at 2016 × 1344. Phantom targets provided by the manufacturer were used to calibrate instrument measurements. Reconstructed samples were analyzed using 3D Slicer (v.5.4.0).

### 4.8. Histology

Dissected, soft-tissue-free specimens were fixed in 2% paraformaldehyde at 4 °C overnight. Samples were decalcified and fixed in a 0.4 M EDTA solution for 3 weeks for mouse specimens and 4 weeks for pig specimens. The specimens were then dehydrated in 30% sucrose at 4 °C overnight. Specimens were embedded in optimal cutting temperature (OCT) compound and sectioned at 10 µm. For histological analysis, representative sections were stained using freshly prepared Movat’s pentachrome, which highlights polysaccharides such as glycosaminoglycans (GAGs) and proteoglycans with blue coloration. Notably, aggrecan, a proteoglycan rich in GAGs, interacts with the stain, resulting in its distinctive blue hue. Aggrecan is a vital component of articular cartilage, providing resistance to friction and compression across joints, and is degraded during the early stages of OA. Select porcine sections were additionally stained with Safranin O/Fast Green, a standard histological stain used to visualize proteoglycan content and cartilage matrix organization.

### 4.9. Immunofluorescence Imaging

Mouse TMJ samples were evaluated by immunofluorescence (IF) for aggrecan, collagen I, and collagen II. Briefly, the OCT embedding medium was removed by washing the sections in PBS. Sections were then incubated with pepsin (2 mg/mL in 0.2 N HCl) for 15 min to expose epitopes, followed by antigen retrieval using Diva Decloaker (Biocare Medical, Pacheco, CA, USA) at 37 °C for 30 min. After blocking with 5% BSA, samples were treated overnight at 4 °C with a 1:100 dilution of the following antibodies: Anti-Collagen I (Abcam, ab34710, Cambridge, UK), Anti-Collagen II (Abcam, ab34712), and Anti-Aggrecan (Abcam, ab216965). Secondary-antibody-only (no-primary) negative control sections were included for each staining run, and antibody specificity was confirmed using known positive-control tissue. After antibody incubation, samples were washed twice with PBS, followed by staining with DAPI (1:1000) for 10 min.

Images were captured using a Nikon Eclipse 50i (Nikon Corporation, Tokyo, Japan). For the murine study, three sections per animal were analyzed, with one field evaluated per section. For the porcine study, ten sections from the merged defect region were analyzed, with two fields evaluated per section. Regions of interest were consistently defined based on anatomical localization of the fibrocartilage and proliferative chondroblastic layers. Investigators were not blinded to group assignment during histological and immunofluorescence image acquisition and analysis.

### 4.10. Histological and Immunofluorescence Image Quantification

Histological and immunofluorescence images were quantified using ImageJ (version 2.14.0). For collagen I, collagen II, and aggrecan immunofluorescence, a staining threshold was established based on no-primary-antibody negative controls and applied to the corresponding images to distinguish positive staining from background. Following thresholding, the area positive for each marker was measured using the ImageJ measurement function. The total cartilage area was defined based on tissue morphology and measured for each image. The percentage of positive staining area (%) was calculated as the marker-positive area divided by the total cartilage area × 100. The relative areas of the surface fibrocartilage layer and proliferative chondroblastic layer were quantified from pentachrome-stained sections using ImageJ. Each layer was identified based on its histological morphology, and its area was measured and normalized to the total cartilage area. Results are expressed as the area of the respective layer divided by the total cartilage area × 100. Image analysis was not performed with the investigator blinded to the experimental group.

### 4.11. Statistical Analysis

Quantitative data from the murine study are presented as mean ± standard error of the mean (SEM). Statistical analyses were performed using GraphPad Prism (version 10.2.2 (341)). Comparisons among the three experimental groups (uninjured control, injury + PBS, and injury + BMP-2/cabozantinib) were performed using one-way ANOVA followed by Tukey’s test. A *p* value < 0.05 was considered statistically significant. Given the small sample size (*n* = 3 per group), normality and homogeneity of variance could not be reliably assessed using formal statistical tests; therefore, the results of the ANOVA should be interpreted cautiously in the context of this pilot study. The porcine study was conducted as a pilot feasibility experiment using a single animal (*n* = 1). Therefore, biomechanical, micro-computed tomography, and histological findings are presented descriptively, and no statistical analyses were performed.

## 5. Conclusions

In summary, our findings demonstrate that combined BMP-2 and cabozantinib treatment is associated with histological changes consistent with TMJ cartilage repair, characterized by qualitative restoration of the fibrocartilaginous and proliferative chondroblastic layers, along with trends toward increased collagen I and aggrecan deposition in a murine TMJ injury model. In addition, we demonstrated a clinically relevant porcine TMJ defect model that enables direct surgical delivery and evaluation of regenerative therapies. Collectively, these findings support continued investigation of BMP-2 combined with cabozantinib as a candidate strategy for TMJ cartilage repair and underscore the importance of restoring the specialized fibrocartilaginous architecture of the TMJ.

## Data Availability

The original contributions presented in this study are included in the article. Further inquiries can be directed to the corresponding authors.
